# The Role of Epstein-Barr Virus in Modulating Key Tumor Suppressor Genes in Associated Malignancies: Epigenetics, Transcriptional, and Post-Translational Modifications

**DOI:** 10.3390/biom12010127

**Published:** 2022-01-13

**Authors:** Adelaide Ohui Fierti, Michael Bright Yakass, Ernest Adjei Okertchiri, Samuel Mawuli Adadey, Osbourne Quaye

**Affiliations:** West African Centre for Cell Biology of Infectious Pathogens (WACCBIP), Department of Biochemistry, Cell and Molecular Biology, University of Ghana, Accra P.O. Box LG 54, Ghana; aofierti@st.ug.edu.gh (A.O.F.); mbyakass@st.ug.edu.gh (M.B.Y.); eaokertchiri@st.ug.edu.gh (E.A.O.); smadadey@st.ug.edu.gh (S.M.A.)

**Keywords:** Epstein-Barr Virus, cancer, tumor suppressor genes, epigenetic, post-translational modifications, EBV nuclear antigens, latent membrane proteins

## Abstract

Epstein-Barr virus (EBV) is ubiquitous and carried by approximately 90% of the world’s adult population. Several mechanisms and pathways have been proposed as to how EBV facilitates the pathogenesis and progression of malignancies, such as Hodgkin’s lymphoma, Burkitt’s lymphoma, nasopharyngeal carcinoma, and gastric cancers, the majority of which have been linked to viral proteins that are expressed upon infection including latent membrane proteins (LMPs) and Epstein-Barr virus nuclear antigens (EBNAs). EBV expresses microRNAs that facilitate the progression of some cancers. Mostly, EBV induces epigenetic silencing of tumor suppressor genes, degradation of tumor suppressor mRNA transcripts, post-translational modification, and inactivation of tumor suppressor proteins. This review summarizes the mechanisms by which EBV modulates different tumor suppressors at the molecular and cellular levels in associated cancers. Briefly, EBV gene products upregulate DNA methylases to induce epigenetic silencing of tumor suppressor genes via hypermethylation. MicroRNAs expressed by EBV are also involved in the direct targeting of tumor suppressor genes for degradation, and other EBV gene products directly bind to tumor suppressor proteins to inactivate them. All these processes result in downregulation and impaired function of tumor suppressors, ultimately promoting malignances.

## 1. Introduction

Epstein-Barr Virus (EBV) is a human herpesvirus composed of a linear double-stranded DNA of approximately 172 kbp. EBV is ubiquitous and is carried by approximately 90% of the world’s adult population [1,2]. The virus exhibits two life cycles, namely, the latent and lytic cycles. In the latent life cycle, the viral genome exists as an episome in the nucleus of the host cell and replicates simultaneously as the host genome replicates. In the lytic replication cycle, the virus encodes for its DNA replication complex to replicate its genome in higher folds, causing the cell to lyse and release the newly formed viruses [3,4,5,6].

EBV has been described to have tropism mainly for nasopharyngeal epithelial cells and B cells [7,8,9], but infection of other cell types, such as natural killer cells, smooth muscle cells, and T cells, have also been reported [8]. While B cells have been suggested to be a reservoir for latent EBV, epithelial cells are a site for lytic replication [8]. The virus has been found to be associated with several malignancies, including gastric carcinoma, Hodgkin’s lymphoma, leiomyosarcomas, lymphoepithelioma-like carcinoma, and nasopharyngeal carcinoma (NPC), with NPC being the most associated [10,11,12]. A comprehensive survey in 2014 reported an estimated 143,000 deaths globally as a result of EBV-attributed malignancies in 2010, representing 1.8% of all cancer deaths [13]. Out of the estimated deaths recorded due to the presence of EBV-related malignancies, gastric cancer and nasopharyngeal carcinoma accounted for 92%, and 47% occurred in East Asia consisting of China, Taiwan, and the Democratic People’s Republic of Korea [13].

Several mechanisms and pathways have been proposed as to how EBV facilitates the pathogenesis and progression of malignancies, the majority of which have been linked to viral proteins that are expressed upon infection including latent membrane proteins (LMPs) and Epstein-Barr virus nuclear antigens (EBNAs) [14]. EBNA-1, one of the first EBV proteins that are expressed upon initial infection and essential for viral DNA replication in the host cell, binds to a specific sequence at the origin of replication of the viral genome to regulate expression of other EBNAs, latent membrane proteins, and other latency genes to facilitate latent infection [15,16]. EBNA-1 directly inhibits phosphorylation of IKK-alpha/beta which results in the inhibition of the canonical NF-_K_B pathway [16], a signaling pathway that is critical in the regulation of cell differentiation, cell growth, and apoptosis and, hence, carcinogenesis [17]. EBNA-1 has also been shown to indirectly modulate the function of p53 to promote cell survival [15]. Another important EBV latency gene product is LMP1; this protein modulates the replication/expression of the EBV genome as well as the host genome [18]. LMP1 is a 63 kDa protein that contains an N-terminal cytoplasmic domain that orients the protein to the plasma membrane, hydrophobic transmembrane loops that allow for oligomerization, and a C-terminal cytoplasmic domain that is involved in signaling [19]. C-terminal activation regions 1 and 2 (CTAR 1 and CTAR 2) are two different functional domains that are distinguished by the difference in their ability to activate the nuclear factor-κB (NF-κB) signaling pathway [20]. LMP1 is involved in the activation of several other pathways, including the signal transducer and activator of transcription (STAT) pathway, the phosphatidylinositol 3-kinase (PI3K) pathway, and the activating protein 1 (AP-1) pathway, to enhance cell proliferation, cell survival, and to suppress apoptosis [18].

This review aimed to summarize the mechanisms by which EBV modulates different tumor suppressors at the molecular and cellular levels in various cancers.

## 2. EBV Associated Malignancies

### 2.1. Burkitt’s Lymphoma

Burkitt’s lymphoma (BL) is a non-Hodgkin’s lymphoma that aggressively affects B cells, mostly in children [21]. It was first identified by Dennis Burkitt in 1958 in Uganda, and cases are still being recorded in Africa, the United States of America, and Europe. The incidence of BL is more prevalent in Eastern Africa and contributed to approximately 20% of childhood cancers in Uganda within the years of 1993–1997 [22]. A recent survey in Africa, in 2018, indicated that BL contributes to approximately 50% of all pediatric cancers (with a total incidence of 3900) and is predominantly reported among children of 14 years of age and below [23].

The World Health Organization (WHO) has classified Burkitt’s Lymphoma into three different categories based on pathological features and geographical locations: endemic Burkitt’s lymphoma, sporadic Burkitt’s lymphoma, and immunodeficiency-associated Burkitt’s lymphoma. Endemic Burkitt’s lymphoma mainly affects African children within 2–14 years of age and occurs in males twice as often as it occurs in females [24,25]. EBV infection and malaria are both associated with the endemic subtype of BL [25]. Sporadic Burkitt’s lymphoma affects the lymph nodes in individuals of all ages and is prevalent in the United States of America and Europe with less than 20% of the cases associated with EBV [25,26,27]. Immunodeficiency-associated Burkitt’s Lymphoma is predominant in HIV patients with CD4 counts usually above 200 cells/μL and may be present in other forms of immunodeficiencies such as patients who have undergone immunosuppressive therapy due to the fact of organ transplant [26]. The immunodeficiency-associated Burkitt’s lymphoma usually affects lymph nodes, bone marrow, and the central nervous system [24].

### 2.2. Nasopharyngeal Carcinoma

Nasopharyngeal carcinoma (NPC) is a tumor of the nasopharynx, and it arises from the epithelial squamous cell [28]. The association of EBV with nasopharyngeal carcinoma was first established in 1966, where NPC patients were found to express antibodies against EBV antigens [29]. NPCs have been reported to be endemic to southern China and Southeast Asia, accounting for approximately 80% of the 65,000 annual cases recorded globally [10,30]. Whereas the lytic phase of EBV predominates in normal epithelial cells, and the latent phase of EBV predominates in epithelial cells in nasopharyngeal carcinoma [10].

Four stages of NPC have been described that include stages I–IV [31]. The Union for International Cancer Control (UICC) and the American Joint Committee on Cancer (AJCC) developed the TNM (tumor, node, and metastasis) staging system to describe the levels of severity within an NPC stage [32]. Stage I describes a small tumor which has neither spread to a lymph node nor has distant metastasis. Stage II describes a tumor in the nasopharynx that has spread to the lymph node in the neck with the presence of EBV in lymph, but the spread is not to the extent of distant metastasis. Stage III describes a large tumor invasive or noninvasive which may spread to the lymph nodes on either side of the neck with no metastasis. Stage IV carcinoma presents an extremely large invasive tumor with lymph involvement with or without extensive metastasis. The NPC staging system is relevant for understanding how to therapeutically approach NPC cases [31].

Several histological classifications of NPC have been proposed over the years, but the most widely used is the classification by the WHO, which according to many sources, has limitations that affect prognosis and treatment [33,34]. WHO classified NPC into three types: squamous cell carcinoma (type I), non-keratinizing carcinoma (type II), and undifferentiated carcinoma (type III) [35]. Whereas types II and III are mostly associated with higher loads of EBV, type I is not.

Many risk factors have been attributed to NPC pathogenesis, which may be environmental, pathogen-mediated, lifestyle, and genetic. A case-referent study conducted in Hong Kong, China, revealed that certain occupations in the craft industry or occupations that expose people to chemical and welding fumes and cotton dust, may put one at risk of NPC [36]. Another study in NPC endemic areas of southern China, conducted among 1049 NPC cases and 785 NPC-free controls who were positive for EBV, also disclosed family history as a risk factor, especially first-to-third-degree relatives, which may account for the genetic factors [37]. The study also reported the risk of certain lifestyle factors that may include high consumption of salted fish, more than 10 years of exposure to domestic wood-cooking fires, and occupational solvents such as acetone, xylene, and formaldehyde. The authors, however, estimated that the family history and environmental factors accounted for a smaller percentage (2.7%) of NPC pathogenesis within high-risk populations [37]. It is widely known that the major risk factor in the development of NPC is EBV infection [38,39,40]; upon latent infection, EBV employs several mechanisms to catalyze the pathogenesis of NPC by expressing type II latency genes.

Certain oncogenic pathways have been implicated in the progression of NPC induced by EBV infection. By activating the NF-κB and Erk1/2 signaling pathways, these EBV-miR-BART8-3p (micro-RNAs) directly target RNT38 in NPC cells, enhancing the progression of NPC by inducing NPC cell migration, invasion, metastasis, epithelial–mesenchymal transition, and expression of NPC metastatic proteins [41]. Other pathways mediated by EBV in NPC include WNT/β-catenin, Janus kinase (JAK)/STAT, PI3K/Akt/mammalian target of rapamycin (mTOR), epidermal growth factor receptor (EGFR), and mitogen-activated protein kinase (MAPK) pathways [42].

Mutations in oncogenes have been reported in NPC patients, but such mutations were not found to correlate with any of the environmental and lifestyle risk factors and were also not frequent among the majority of NPC cases [43]. In an attempt to determine if mutations in p53 facilitate the development of malignant clones in NPC, several types of mutations including single-point mutation, frameshifts, deletions, and duplications were observed to be highly frequent among nude mouse-passaged tumors, and very less frequent among metastatic and primary tumor, indicating that p53 mutations are less likely to mediate initial pathogenesis of clonal outgrowth of NPC [44]. EBV, therefore, harnesses the mechanisms involved in regulating oncogene expression and protein functions, such as epigenetics, transcription, translation, and post-translational modifications, to deregulate the expression and function of the various oncoproteins that facilitate NPC pathogenesis in ways that are later discussed in this paper.

### 2.3. T-Cell Lymphoma

T-cell non-Hodgkin’s lymphomas (NHLs) are rare malignancies that makeup approximately 12% of all lymphomas [45], and they are mostly characterized by extranodal disease and necrosis or apoptosis in biopsy samples [46]. T-cell lymphomas (TCLs) are grouped into two main subtypes: peripheral T-cell lymphomas (PTCLs) and cutaneous T-cell lymphomas (CTCLs). All TCL subtypes have distinct characteristics and require specific diagnostic and therapeutic treatment strategies; the wide range of subtypes with varied clinical outcomes makes the study of the disease quite challenging [47].

PTCLs are rare cancers, associated with poor prognosis [48], with few subtypes known to have an association with EBV [49], and makeup approximately 26% of all mature T-cell and natural killer cell neoplasms [50]. Males are at a higher risk for most PTCL subtypes [51]. Some risk factors for PTCL include smoking, alcohol consumption, a history of eczema, or a genetic history of hematologic cancers [52]. Wang et al. further noted that there was a correlation between reduced risk of PTCL and patients with allergies or living or working on a farm [51].

Angioimmunoblastic T-cell lymphoma (AITL) is a common subtype of PTCL, and the pathology of the lymphoma has been associated with infectivity of EBV [53]. However, the exact interaction between EBV and AITL remains largely unknown. Delfau-Larue et al. (2012) [54] showed a correlation between circulating EBV DNA and the presence of circulating AITL malignant cells, and higher levels of EBV DNA at initial presentation correlated with poorer response to treatment [53].

Epigenetic modulations, such as histone modification and DNA methylation, have been observed in TCL [55]; however, epigenetic modulation of EBV proteins and microRNAs on TCL remains largely unknown [56] and, therefore, requires extensive research.

### 2.4. Gastric Cancers

Gastric cancer is the second cause of death resulting from cancer and ranks fourth among all cancer incidents worldwide [57]. Out of the annual gastric cancer cases of about 990,000 globally, approximately 780,000 patients lose their lives [58]. Factors that have been found to increase the risk of gastric cancer include diet, smoking, alcoholism, family history, and infections with *Helicobacter pylori* and Epstein-Barr virus [57,59]. The Cancer Genome Atlas (TCGA) grouped gastric adenocarcinomas into four main categories: EBV-associated, microsatellite instability, chromosomal instability, and genomically stable gastric carcinomas [60]. EBV-associated gastric cancers account for 8.8% of all gastric cancer cases [61] and are thought to result from EBV latent infection that causes abnormalities, such as aberrant host DNA methylation [62] with the expression of the EBV latent gene, LMP2A, in the majority of cases [63].

### 2.5. Breast Cancers

Breast cancer is the most diagnosed malignancy and the leading cause of cancer mortality among women in the world. According to estimates by the WHO, 2.1 million women are diagnosed with breast cancer annually. Approximately 627,000 women died from the disease globally in 2018, accounting for approximately 15% of all deaths caused by cancer in women [64]. Risk factors of breast cancer include age, family history, menarche, delayed menopause, first pregnancy after 25 years of age, nulliparity, long-term consumption of exogenous estrogens, obesity after menopause, and encountering ionizing ray [65]. EBV has also been found to be an etiological reason for breast cancer [66]; the virus infects mammary epithelial cells and has been detected in breast tissues and in human breast milk [67]. The first positive association of EBV infection with breast cancer was reported in 1995 [68], after which multiple studies have been performed to investigate the link between EBV and the pathogenesis of breast cancer. For example, it has been suggested that EBV infection makes breast epithelial cells susceptible to malignant transformation through activation of the HER2/HER3 signaling pathway [69]. On the other hand, some studies have reported a lack of association between EBV and breast cancer [70,71]. More so, the detection of EBV in human breast cancer biopsies tends to show some geographic bias [72] and, hence, a general conclusion on a causative attribute of EBV in breast cancers cannot be clearly made.

## 3. Key Oncoprotein and Tumor Suppressors Involved in EBV-Associated Malignancies

Tumor suppressor genes are involved in the repair of mistakes in DNA during replication, regulation of cell division, and apoptosis, and the improper functioning of these genes leads to different malignancies [73]. Summarized in Table 1 are EBV modulators that are associated with different malignancies.

### 3.1. Proto-Oncogene/Oncogene Associated with EBV-Associated Burkitt’s Lymphoma

#### c-Myc

Burkitt’s lymphoma is characterized by dysregulation of the *c-Myc* gene resulting from c-Myc translocation into the loci of the immunoglobulin gene [75,76]. c-Myc is a transcription factor and a DNA binding protein that when activated targets the binding of enhancer box DNA sequences, regulating the expression of host genes [75,77]. The *c-Myc* gene has two promoters: P2, which is the predominant promoter and facilitates the transcription of the *c-Myc* gene; P1, which also facilitates transcription but mostly after translocation of the gene, where transcription is under the influence of an immunoglobulin enhancer element [78,79]. Pathways, such as PI3-K, which modulates c-Myc activity, have been implicated in molecular and genetic pathogenesis of BL and may facilitate the regulation of cell division and proliferation of cancer cells [75]. These pathways, however, are highly vulnerable to EBV infections in ways that enhance the maintenance and progression of Burkitt’s lymphoma cells.

### 3.2. Tumor Suppressor/Oncogene Associated with Nasopharyngeal Carcinoma

#### 3.2.1. p53

Overexpression of mutant p53 has been implicated in the development of NPC following EBV infection [80]. p53 is a DNA-binding protein and a transcription factor that facilitates the activation of several genes in response to stimuli such as DNA damage and oncogene activation [81]. This protein induces cell-cycle arrest and allows for apoptosis to occur, which is prioritized in the treatment of cancer for killing damaged or cancer cells [82]. In addition, p53 helps to maintain chromosomal integrity in cell-cycle checkpoints through cell-cycle arrest, which allows for double-stranded break repair [83].

#### 3.2.2. E-Cadherin

E-cadherin is a tumor suppressor and a calcium-dependent cell adhesion molecule that plays a major role in cell–cell adhesion in epithelial tissues [84]. Loss of the protein affects the morphology of epithelial cells and promotes metastasis in malignant cells [85]. Compared to normal tissues, E-cadherin expression was observed to be reduced in tissues from NPC patients, suggesting that the protein plays a role in the invasion and metastasis of cancer [86]. Though the mechanism is not fully understood, E-cadherin is inhibited by miR-BART9, an EBV miRNA, to stimulate the formation of a mesenchymal-like phenotype and, subsequently, promotes migration of NPC cells [87]. Migration of NPC cells is favored by downregulation of the cadherin–catenin cell adhesion complex [88].

### 3.3. PD-L1 Tumor Suppressor/Oncogene Associated with T-Cell Lymphoma

Programmed cell death ligand 1 (PD-L1) has been implicated in several malignancies including T-cell lymphoma, and has become a promising therapeutic target [89,90]. PD-L1 is a key regulator of T-cell-mediated immune response against malignancies [91]. The binding of PD-L1 to its receptor, programmed cell death receptor 1 (PD-L1), blocks T cells from eliminating PD-L1-containing cells, including tumor cells [92]; therefore, making PD-L1 a potential tumor suppressor [74].

## 4. Different Ways That EBV Genes/Gene Products Modulate Tumor Suppressors

### 4.1. Modulation of c-Myc in BL and Its Effect on Transcription

#### 4.1.1. The Role of EBNA-2

**Figure 1:**
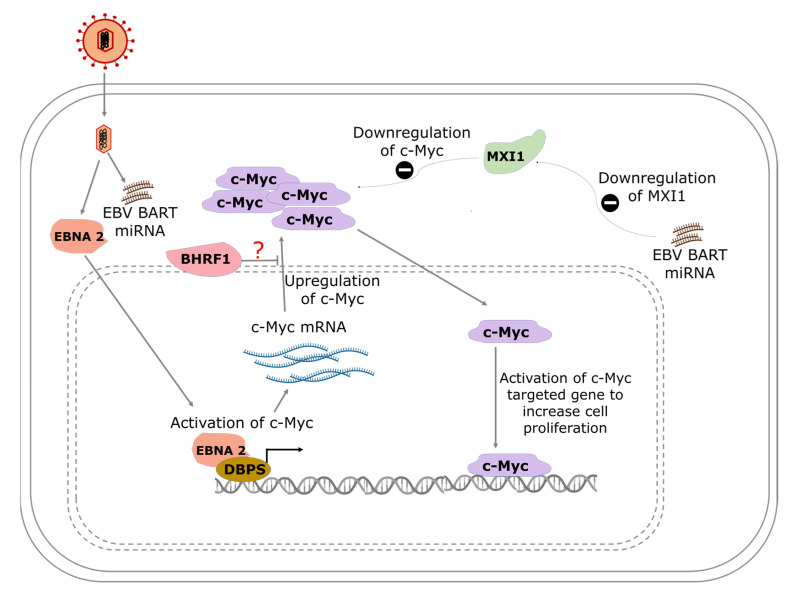
A model showing the pathways of EBV-mediated c-Myc activation in Burkitt’s lymphoma. EBNA-2 interacts with albumin D box-binding proteins (DBPs) to activate the transcription of c-Myc. Increased production of c-Myc results in the activation of the c-Myc-targeted gene to increase cell proliferation. EBV BART miRNA inhibits MAX interactor 1 (MXI1), which is known to repress the activity of c-Myc.

Aside from the role of EBNA-2 in facilitating B-cell immortalization, it also serves as a transcription activator that regulates both viral and host genes. Proto-oncogene c-Myc is directly activated and upregulated by EBV gene product EBNA-2 (Figure 1). To understand the modulation of c-Myc by EBNA-2, Kaiser et al. generated a construct where EBNA-2 was fused with estrogen receptor (ER-EBNA-2) [93]. ER-EBNA-2 was activated by estrogen stimulation. It was shown that the regulation of c-Myc expression occurs at the level of transcription and was observed when c-Myc RNA levels increased sharply over 2 h in the presence of cycloheximide, a translation inhibitor, as opposed to a gradual increase over 6 h in the absence of the inhibitor [93]. The increased c-Myc expression in the presence of the inhibitor of de novo protein synthesis may be due to the preexisting inactive EBNA-2, which was activated upon estrogen treatment. While EBNA-2 may be playing a key role in the activation of c-Myc, and earlier findings have shown that c-Myc activation can occur independently of EBNA-2 [94].

Studies have shown that EBNA-2 interacts with other viral proteins to exert its transcriptional activation or regulatory effect on host genes. The ability of EBNA-2 to transactivate LMP1 promotor in order to regulate expression is mediated by J kappa, a DNA-binding protein in the cell, and PU. 1, a transcription factor that binds to specific DNA sequences [95,96]. EBNA-2 also regulates c-Myc by utilizing enhancer interactions that are −168 to −186 of the MYC transcription start site, and these elements are rich in EBNA-2 and recombination signal binding protein for immunoglobulin kappa J (RBPJ) signals [97].

#### 4.1.2. Regulation of c-Myc by EBV-Encoded MicroRNAs

MicroRNAs (miRNAs) are small non-coding RNAs made of approximately 17–23 nucleotides that are obtained from the non-coding region of the human genome [98]. The role of miRNAs have been implicated in several diseases and cancers including Alzheimer’s disease, lung cancer, and gastric and pancreatic cancers; hence, they are receiving attention for repurposing as potential biomarkers for diseases such as Parkinson’s disease [99], cardiovascular diseases [100], diabetes mellitus [101], and Alzheimer’s disease [102]. miRNAs function by binding to the 3′ untranslated region of mRNA, leading to degradation of the mRNA and repression of translation [103,104]. As such, miRNAs have the ability to greatly influence the regulation of cellular processes such as cell growth and differentiation [104]. In cancers, the expression of miRNAs are dysregulated [105].

miRNAs are also present in microorganisms, including viruses [106] and miRNA BamHI-A rightward transcripts (BARTs), also referred to as complementary strand transcripts, and they have been shown to suppress the expression of lytic genes in EBV-infected cells to enhance viral latency [107]. The EBV miRNAs are transcribed either as BART-A miRNA or as BamHI fragment H reading frame 1 (BHRF1), the majority of which are expressed as BART-A miRNA [108]. miRNA BARTs are EBV single-stranded, non-coding messenger RNAs that are involved in post-translation regulation of both EBV and host gene expression [108]. Six different splicing forms of EBV miRNA BART have been identified and some have been found to be upregulated in response to EBV reactivation [109]. EBV latent infection has been shown to regulate several host genes that are involved in proliferation, apoptosis, cell survival, and gene expression. Studies of the AGS cell line have shown that 53 and 101 genes were upregulated and downregulated, respectively, by approximately two-fold following latent EBV infection [40].

Using a Myc-responsive luciferase reporter, the ability of EBV BART miRNA to activate the MYC pathway was demonstrated during EBV type-I latency after prediction with bioinformatics tools [40]. The study also found that Max-interacting protein 1 (MXI1), a protein that negatively regulates MYC activation (translocation), was downregulated and partially accounted for the activation and increased activity of MYC following EBV infection (Figure 1) [40].

#### 4.1.3. Epigenetic Effect of EBV Modulation of c-Myc

EBV-induced epigenetic effect becomes a priority in the pathogenesis of BL, having been discovered that EBV-positive BL has fewer mutations compared to EBV-negative BL [110]. Both EBV miRNA and MYC have been found to contribute to the regulation of miRNA-29 in BL cells [111]. Inhibition of EBV-miR-BART6-5p altered the expression of miRNA-29 in BL cells, whereas miRNA-29 expression was regulated by MYC through epigenetic methylation of the promoter and enhancer sequences of miR-29a/b1 and miR-29b2/c genes.

Akata BL cells without the EBV genome were used to demonstrate that EBV contributes to tumorigenesis in BL cells by regulating the expression of c-Myc [112]. When type-I latency was re-established in the EBV negative Akata cells, tumorigenesis was restored, and an increased resistance to apoptosis under growth limiting conditions was observed. Higher levels of Bcl-2 as well as an EBV-dependent decrease in c-Myc proteins were associated with the observed anti-apoptotic effect of EBV [112].

### 4.2. Modulation of p53 and E-Cadherin in Nasopharyngeal Carcinoma

#### 4.2.1. The Role of miRNAs in EBV-Induced Transcriptional Regulation of p53

EBV-miR-BART5-3p targets the 3′-untranslated regions of p53, suppresses expression, and significantly decreases p53 mRNA [113]. Aside from targeting mRNA, EBV-miRNA5-3p also facilitates the degradation of the p53 protein, a process that leads to the inhibition of cell cycle arrest and apoptosis and enhances the progression of NPC [113].

#### 4.2.2. The Role of EBNA 3C in EBV-Induced Post-Translational Modification of p53

**Figure 2:**
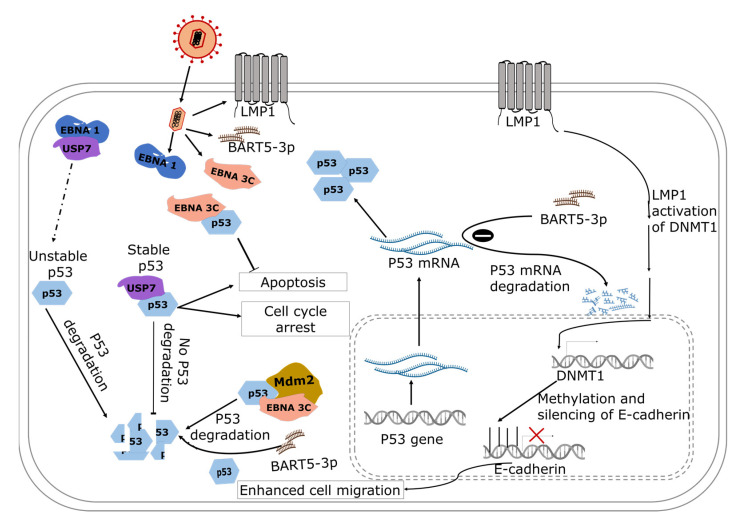
A model showing the role of EBV gene products in the regulation of nasopharyngeal carcinoma. EBNA-1 interacts with USP7 and indirectly reduces the stability of p53, and the unstable p53 is degraded. EBNA-3 binds with p53 to inhibit apoptosis and favors cell proliferation. In addition, BART5-3p is involved in the degradation of p53 mRNA and downregulates the expression of p53. The broken arrow represents the indirect activity of EBNA1 resulting in unstable p53. The solid arrows show the direction of the associated pathways.

The N-terminal domain of EBNA 3C binds directly to the C-terminal DNA-binding domain of p53, suppressing the apoptotic and transcriptional activities of the oncoprotein [114]. The C-terminal domain regulates the sequence-specific binding of core p53, and non-specific binding of double-stranded DNA to the C-terminal domain has been shown to inhibit sequence-specific binding of the core domain of p53 [115]. The data suggest that dysregulated binding of the C-terminal domain can impair the function of p53 (Figure 2).

Studies have shown that EBNA 3C recruits the SCFSkp2 E3 ubiquitin ligase complex to enhance the degradation of regulators of the cell cycle including retinoblastoma and p27KIP [114,116]. The C- and N-terminals of EBNA 3C possess deubiquitinating activity and can induce self-deubiquitylation or deubiquitinates murine double minute 2 (Mdm2) to produce a stable Mdm2. Coimmunoprecipitation experiment targeting EBNA 3C confirmed the formation of stabilized ternary complex, where EBNA 3C uses its N-terminal domain to bind simultaneously to human Mdm2 and p53 [117]. The authors showed that EBNA 3C promotes the degradation of p53 indirectly by (1) stabilizing Mdm2 and (2) enhancing the E3 ligase activity of Mdm2. In addition, EBV can induce degradation of phosphorylated p53 independent of Mdm2 by harnessing a ubiquitin–proteosome pathway that is induced during the lytic stage of viral replication where DNA damage occurs [118] (Figure 2).

#### 4.2.3. EBV-Induced Post-Translational Modification of p53: The Role of EBNA1

EBNA1 binds competitively with high specificity to ubiquitin-specific protease 7 (USP7) at the same site where USP7 binds to stabilize p53 [15] and, as a result, p53 is more likely to remain ubiquitinated, making it unstable. With respect to NPC cells, a decrease in p53 levels in NPC cells that are expressing EBNA-1 compared to non-expressing cells has been observed, suggesting that the interference of EBNA-1 in USP7 interaction with p53 could suppress apoptosis [119].

#### 4.2.4. The Role of LMP1 in EBV-Induced Post-Translational Modification of p53

Contrary to the degradative effect of EBNA1 and EBNA3C on p53, LMP1 accumulates and phosphorylates p53 [120]. LMP1 interacts with tumor necrosis factor receptor-associated factor 2 (TRAF2) to increase p53 accumulation via K63-linked ubiquitination while suppressing E3 ligase–MDM2-mediated ubiquitination [120]. These processes allow LMP1 to modulate the function of p53 by suppressing apoptosis and preventing cell cycle arrest induced by p53 [121,122].

### 4.3. EBV-Induced Epigenetic Modulation of E-Cadherin in Nasopharyngeal Carcinoma

#### The Role of LMP1

LMP1 activates DNA methyltransferase 1, which hypermethylates CDH-1 promoter and, subsequently, leads to silencing of the expression of the E-cadherin gene [123]. By activating DNA methyltransferase 1, LMP1 activates the c-Jun N-terminal activator protein-1 (JNK-AP-1) pathway and the DNMT1-promoter 1 (P1), inducing the formation of DNMT1 and the histone deacetylase-containing transcriptional repression complex at the E-cadherin gene promoter [124]. The downregulation of the E-cadherin gene also induces cell migration, which facilitates the progression of NPC. Whole-genome profiling of NPCs has also provided evidence from clinical tumor samples on immune escape of NPCs using host and virial processes [125]. Constitutive activation of NF-κB due to the LMP1 expression and genomic aberrations was observed in 90% of NPCs. The genomic aberration and the expression of viral genes was postulated as a possible mechanism by which NPCs evade the immune system [125].

### 4.4. Modulation of PD-L1 and c-Myc in T-Cell Lymphoma

#### The Role of LMP1

A significantly higher expression of PD-L1 at both mRNA and protein levels in SNK-6 (EBV-positive) cells compared to NK-92 (EBV-negative) cells have been reported, and a positive correlation between EBV oncoprotein, LMP-1, and PD-L1 was identified [112]. The upregulation of PD-L1 by LMP-1 is mediated through the MAPK/NF-κβ pathway in TCL [126]. Upon EBV infection, EBNA2 and LMP-1 viral proteins target c-Myc at the transcription level, thereby causing c-Myc upregulated expression that eventually leads to the constitutive transcription of surviving—an antiapoptotic molecule [127,128]. The proliferation and overexpression of LMP-1 is postulated to be induced by the actions of IL-2, IL-9, and IL-10 [129].

### 4.5. EBV Modulates Tumor Suppressor Genes in Gastric Cancers

#### 4.5.1. The Role of LMP2A

EBV-induced methylation is the most common modulation in tumor suppressor genes in gastric cancers [60], but the exact mechanism is not fully understood. While LMP2A can activate DNA methyltransferase 1 (DNMT1) through STAT3 phosphorylation, it may not be expressed in all epithelia cells of gastric cancers [130]. Methylation of both viral and host DNA are important for the pathogenesis of EBV-associated gastric cancers [131]. In the viral genome, methylation occurs in the latent genes, suppressing their expression and allowing the virus to escape the attack from cytotoxic T lymphocytes. In the host genome, tumor suppressor genes, including CDKN2, CDH1, p15, and p73 PTEN, are targeted for methylation, which occurs at the CpG site. LMP2A expression has also been found to upregulate the expression of DNMT3b which correlates with hypermethylation of several gene promoters [132,133].

#### 4.5.2. The Role of EBV-Encoded miRNAs

Elevated levels of miR-BART20-5p were found to be associated with worst survival in patient with EBV-associated gastric cancers [134]. The EBV miR-BART20-5p directly targets the 3′ untranslated region (3′ UTR) of Bcl2-associated agonist of cell death (BAD), an apoptosis-inducing factor that facilitates apoptosis, leading to a significant reduction in BAD cellular mRNA and protein levels [135]. miR-BART20-5p was observed to suppress apoptosis and enhance cell growth, suggesting its contribution in the development and progression of gastric cancers. miRNA-BART10-3p have also been reported to promote cell proliferation and migration in EBV-associated gastric cancers by targeting and degrading a member of the Dickkopf protein family, DKK1, which is a host gene involved in embryonic development and a known antagonist of the WNT signaling pathway [136]. Another EBV miRNA thought to promote tumorigenesis in gastric cancers is miRNA-BART1-3p, which has been observed to degrade and suppress the levels of E2F3, a cell cycle regulator [137].

### 4.6. EBV Modulates Tumor Suppressors in Breast Cancer

#### The Role of EBNA 3C

EBNA 3C binds directly to nucleoside diphosphate kinase non-metastatic clone 23, isoform H1 (Nm23-H1) and downregulates the enzyme to facilitate cancer cell survival and progression [138]. Nm23-H1 is the metastasis suppressor that phosphorylates the kinase suppressor of Ras to suppress MAPK signaling, leading to a downstream effect of inhibiting cancer cell proliferation [139]. Although EBV has been detected in breast cancer biopsies, its contribution to pathogenesis or progression of breast cancers remain unclear and a controversy [72].

## 5. Expert Comments

EBV infection is highly ubiquitous, with over a 90% incidence rate globally, but it is intriguing that EBV lies “dormant” in the latent infection stage in most infected people. The need to understand the specific triggers of EBV re-activation in people with EBV-induced malignancies therefore requires further investigation, and it cannot be overemphasized. The key EBV proteins, LMP1 and LMP2, which are known to drive initiation and progression of EBV-induced carcinomas, may offer therapeutic potential as druggable targets to stop or regress carcinomas. Indeed, this concept has been explored experimentally by employing affibodies (small affinity proteins of approximately 6.5 kDa) that are capable of inhibiting the phosphorylation of AKT and thereby blocking the nuclear translocation of b-catenin and eventually terminate c-Myc oncogene expression [140].

The specific host defense mechanisms that keep EBV in latent infection and prevent reactivation in asymptomatic EBV-infected people require further investigation. A clear understanding of such mechanisms will guide the rational development of preventive and therapeutic interventions against EBV-induced malignancies.

Questions yet remain about the specific contribution of environmental triggers or microbiome indicators for EBV reactivation in people with EBV-induced malignancies. Preliminary studies have indicated exposure to solvents, such as acetone and consumption of salted fish, as possible contributors to some EBV-induced malignancies [141,142]. Such claims need to be further investigated and validated to proffer concrete healthcare and policy directions.

## 6. Conclusions

The key protooncogenes and tumor suppressors identified in various EBV-associated malignancies are c-Myc, p53, E-cadherin, and PD-L1. The viral proteins EBNA 2, EBNA 3C, LMP1, and EBV BART5 miRNA were found to modulate host proteins in associated malignancies. This review discussed the different molecular mechanisms by which EBV modulates different tumor suppressors and serves as comprehensive literature to inform future research.

## Figures and Tables

**Table 1:** Tumor suppressor/oncoprotein and EBV modulators in various cancers.

Cancer	Tumor Suppressor/Oncoprotein	EBV Modulators
Burkitt’s lymphoma	c-Myc	EBNA-2
		EBV-encoded micro-RNAs
		BHRF1
Nasopharyngeal carcinoma	p53	EBV-miR-BART5-3p
		EBNA 3C
		EBNA1
		LMP1
		EBNA2
	E-cadherin	LMP1
T-cell lymphoma	PD-L1 [74]	LMP1
Gastric cancers	PTEN, CDKN2, CDH1, p15, p73, etc.	LMP2A
		EBV-encoded microRNAs
Breast cancer	Nm23-H1	EBNA 3C

## Abbreviations

EBV	Epstein-Barr virus
LMPs	Latent membrane proteins
EBNAs	Epstein-Barr virus nuclear antigens
NPC	Nasopharyngeal carcinoma
IKK	Inhibitory-κB kinase
NF-κB	Nuclear factor kappa B
CTARs	C-terminal activation regions
STAT	Signal transducer and activator of transcription
PI3K	Phosphatidylinositol 3-kinase
BL	Burkitt’s lymphoma
UICC	Union for International Cancer Control
AJCC	American Joint Committee on Cancer
TNM	Tumor, node, and metastasis
JAK	Janus kinase
mTOR	Mammalian target of rapamycin
EGFR	Epidermal growth factor receptor
MAPK	Mitogen-activated protein kinase
NHLs	Non-Hodgkin’s lymphomas
TCLs	T-cell lymphomas
PTCLs	Peripheral T-cell lymphomas
CTCLs	Cutaneous T-cell lymphomas
AITL	Angioimmunoblastic T-cell lymphoma
PD-L1	Programmed death-ligand 1
PTEN	Phosphatase and tensin homolog
CDKN2	Cyclin-dependent kinase inhibitor 2
Myc	Master regulator of cell cycle entry and proliferative metabolism
Bcl-2	B-cell lymphoma 2
Mdm2	Mouse double minute 2 homolog
USP7	Ubiquitin-specific-processing protease 7
TRAF2	Tumor necrosis factor receptor-associated factor 2
DNMT1	DNA methyltransferase 1
DKK1	Dickkopf protein family
